# Synergistic approach of structure-based and ligand-based drug design for the development of selective cannabinod receptor ligands

**DOI:** 10.1186/1758-2946-4-S1-P11

**Published:** 2012-05-01

**Authors:** Robert Günther, Peter Brust

**Affiliations:** 1 Institute of Radiopharmacy, Helmholtz-Zentrum Dresden-Rossendorf, Research Site Leipzig, Leipzig, 04318 Germany

## 

Cannabinoid receptors (CB) are G-protein coupled receptors involved in many physiological processes, like learning, appetite, nociception and others. Two subtypes (termed CB1 and CB2) are involved in slightly different processes [1]. Thus, it is important to gain more insight into the the cannabinoid receptor system and the potential effects of cannabinoid therapeutics.

By combining [2] 3D-QSAR, pharmacophore modeling, comparative modeling and molecular docking we could identify features responsible for receptor subtype specificity.

Various pharmacophore models were derived from in-house libraries and data available in the literature. 3D structures of both receptor subtypes were created employing comparative modeling methods. The models were subjected to molecular simulations in solvated lipid bilayers to sample different receptor conformations. The models were used for molecular docking studies with small compound libraries. Employing the data obtained in the pharmacophore/3D-QSAR studies as additional constraints delivered valuable information on affinity and selectivity of the compounds towards CB1 and CB2. The results from this synergistic modeling approach could improve our understanding of the protein–ligand interactions involved.

## References

[B1] Pertwee RG Ligands that target cannabinoid receptors in the brain: from THC to anandamide and beyond Addict Biol 2008 13 147 159 10.1111/j.1369-1600.2008.00108.x 18482430

